# Adaptive Real-Time Object Detection for Autonomous Driving Systems

**DOI:** 10.3390/jimaging8040106

**Published:** 2022-04-11

**Authors:** Maryam Hemmati, Morteza Biglari-Abhari, Smail Niar

**Affiliations:** 1 Department of Electrical, Computer, and Software Engineering, The University of Auckland, Auckland 1010, New Zealand; m.abhari@auckland.ac.nz; 2 Institut National des Sciences Appliquées (INSA) Hauts-de-France, Université Polytechnique Hauts-de-France, 59300 Valenciennes, France; smail.niar@uphf.fr

**Keywords:** real-time detection, hardware accelerator, partial reconfiguration, pedestrian detection, vehicle detection, FPGA, Adaptive ADS, HOG, SVM, DBN

## Abstract

Accurate and reliable detection is one of the main tasks of Autonomous Driving Systems (ADS). While detecting the obstacles on the road during various environmental circumstances add to the reliability of ADS, it results in more intensive computations and more complicated systems. The stringent real-time requirements of ADS, resource constraints, and energy efficiency considerations add to the design complications. This work presents an adaptive system that detects pedestrians and vehicles in different lighting conditions on the road. We take a hardware-software co-design approach on Zynq UltraScale+ MPSoC and develop a dynamically reconfigurable ADS that employs hardware accelerators for pedestrian and vehicle detection and adapts its detection method to the environment lighting conditions. The results show that the system maintains real-time performance and achieves adaptability with minimal resource overhead.

## 1. Introduction

Autonomous driving systems (ADSs) will be used more widely when their use on the roads is legislated. ADS’ legal implications are mostly related to their reliability and safety concerns, which could be addressed when these systems guarantee reliable actions in the case of any hazardous situation. The safety-critical nature of ADS imposes hard real-time requirements on the system. Real-time systems are responsible for completing their task within a specified period; otherwise, it is considered a failure. Hard real-time systems, also known as immediate real-time systems, should be able to complete their operation within the stringent deadline. While missing a deadline in soft real-time systems may lead to a significant loss, it would be catastrophic in hard real-time systems. A simple example of such hard real-time applications is the anti-lock brakes in a car. Time constraints associated with hard real-time applications add more complexity to the design of these systems.

ADSs are expected to provide safer and more reliable driving than human drivers. Accurate obstacle detection is an essential prerequisite to making a reliable decision in these autonomous systems, which results in the need for several complicated and advanced object detection algorithms. There are various obstacles, and they could appear on the road in different environmental conditions. A change in environment could affect the appearance of objects and make the detection task more challenging. All these variations should be considered for robust detection in a system designed for a safety-critical application such as ADSs. Detecting variants of obstacles in different environments and lighting conditions requires extra trained models and more computation during the detection phase. These additional requirements result in more challenging resource allocations for real-time applications where resource-aware and energy-efficient merits play a significant part in design decisions.

We look into the problem of pedestrian and vehicle detection in ADSs. Our design targets real-time detection of pedestrians at different sizes and distances and car detection in various lighting conditions for images of 1920 × 1080. To address the challenge of vehicle detection in different environmental conditions, we consider three different scenarios of day, dusk and dark. We base our design on our previous works where a pedestrian detection hardware accelerator was presented by introducing a specific memory hierarchy [1] and algorithmic modification of the detection algorithm [2]. We also use the models that were developed in our previous work [3] for vehicle detection for three different lighting conditions and build our adaptive system on top of them. We deploy four ARM Cortex A53 processors on the Zynq UltraScale+ development kit to manage the data transfer between the software and hardware accelerators and initiate and monitor the process of dynamic reconfiguration. The vehicle detection models and algorithms are updated through dynamic and partial reconfiguration while pedestrian detection runs uninterrupted.

Considering that training and using different models for detecting an object in different environmental conditions could result in better performance and exploiting the unique reconfiguration feature of FPGA platforms, we develop a system that targets adaptive vehicle detection. The main contribution of this work is creating the framework on a heterogeneous computing platform where several hardware accelerators are deployed on programmable logic (PL) and are coordinated through the ARM processors on the processing system (PS). With our partial reconfiguration approach, we deploy three different algorithms by utilizing the hardware resources required for only one algorithm. We use the partial reconfiguration controller developed in our previous work [3] to minimize the time overhead for the reconfiguration process. However, even during the reconfiguration process, pedestrian detection is not interrupted. Our partial reconfiguration approach results in added adaptability to the system with negligible increased hardware resources.

The rest of the paper is organized as follows. A review of pedestrian and vehicle detection methods is provided in Section 2. Section 3 presents our adaptive system design and the task division between the hardware and software. The pedestrian and vehicle detection algorithms, training and test datasets, and the accuracy of trained models are discussed in Section 4. The hardware implementations of the accelerators and their performance are provided in Section 5. Section 6 provides the hardware resource utilization and an evaluation of the hardware implementations by comparing their processing time with the required processing time of the same algorithms on the PS part of the Zynq UltraScale+ platform. The advantages of partial and dynamic reconfiguration in providing system adaptability with minimal resource overhead are also discussed. Concluding remarks are provided in Section 7.

## 2. Literature Review and Background

During the last few decades, various detection algorithms have been proposed and evaluated for object detection. These methods can be divided into different categories of shape-based detection, motion-based detection and a combination of shape and motion-based detection [4]. They range from conventional machine learning (ML) approaches [5,6,7,8,9] to deep learning (DL)-based techniques [10,11,12]. In conventional ML approaches, the features are extracted and described through some human-defined algorithms and are passed through a classification stage for the final decision. However, in the DL approach, both feature extraction and classification stages are managed within the network, and it is only the art of designing suitable network architectures so that the required features could be extracted and distinguished efficiently.

Several algorithms are developed in the context of conventional ML. However, most of them rely on a few well-known feature descriptors with some modification in either the extraction algorithm or classification structure. One of these early developed features is Haar-like features where a wavelet template is used to define the shape of an object in terms of a subset of the wavelet coefficients of the image [13]. Cascades of Haar-like features proposed by Viola and Jones in 2001 [5] are another early method used for object detection. Originally developed for face detection, this method has the advantage of low computation by introducing a new image representation called “integral image” and yields the detection rate of 15 frames per second (fps) in the original work of face detection [5]. A modified version of the AdaBoost classifier is used for the classification purpose in this study [6]. This approach is tailored to human detection by taking into account the motion information by Viola and Jones in 2003 [7].

Histogram of oriented gradients (HOG), introduced by Dalal and Triggs in 2005, is the other well-known early approach in human detection [8]. HOG features are considered one of the most efficient and promising features for human detection within the context of conventional machine learning approaches where the features are handcrafted. These features are usually used in conjunction with a classifier such as AdaBoost [6] or a support vector machine (SVM) [9] to detect the specific class of objects [14]. HOG features have also been employed in the detection of other objects, such as vehicles, and have shown reasonably accurate detection results compared to other traditional vehicle detection algorithms [15].

Several research works have studied the detection of cars either based on its appearance, its motion or a combination of both [15]. While motion-based methods look at the sequence of frames and employ both detection and tracking algorithms [16,17], appearance-based detection mainly relies on the pixel information of one image frame. In general, these methods extract vehicle appearance features and compare them with a pre-trained model through the classification stage. Various visual features of vehicles are used for this purpose, including the overall shape of car, edges, corners, underneath shadow of the vehicle, headlight or taillight position, and their color. However, all of these features are somehow affected by the environmental condition, which makes the challenge of accurate detection even more complicated.

Deep learning (DL) algorithms have become more popular due to the increased data volumes and improvements in computing power and storage. Modern approaches for object detection are mostly based on deep neural networks (DNNs) and convolutional neural networks (CNNs). In these approaches, the features are not extracted by a human. Instead, the first few layers of the networks are meant to extract the features and build up more complicated features through the network layers. The final layers of the network act as the classification stage to make the final decision and classify the image into several categories.

CNNs are mostly used in image processing, where the massive number of input pixels results in a huge amount of calculations in feed-forward DNNs. The idea behind these architectures is that within the image, nearby pixels constitute features of the objects and applying convolution filters to the pixels could effectively extract these features. CNNs consist of a series of convolutional layers where each convolutional neuron processes the data only within its receptive field [18]. While CNNs are a good option for image classification, applying them to object detection problems results in huge computations. Detecting an object within an image requires several regions of the image to be examined at different scales, which make CNNs inefficient for object detection tasks. Architectures such as region CNN (R-CNN) [19], Fast R-CNN [20], Faster R-CNN [21], you only look once (YOLO) [22] and some other lightweight variations of CNN [10,11,12] have been developed to address this issue and bypass the problem of including too many regions.

Deep belief networks (DBNs) are a stack of separately trained restricted Boltzmann machines (RBMs) [23] layered on top of each other. RMBs consist of one input layer, one output layer and few hidden layers in between. Each hidden layer is considered as the visible layer to the next layer RBM. There are no connections within a layer, and the nodes of each layer are only connected to their next layer [24].

CapsNets are the most recent type of NN architectures with the capability of modeling spatial relationships. While CNNs could extract the features of objects, the location information between the features is not kept within the layers. Missing the location information could result in incorrect classification where the object parts are available but are in the wrong order. CapsNets address this failure of CNNs by introducing the concept of a capsule. Capsules are the nested set of a few neurons in a layer and output a vector to represent the existence of the entity [25].

## 3. Adaptive System Design

Pedestrian and vehicle detection are the two main tasks of an ADS. While there are many other obstacles on the road that these advanced systems should detect, we consider the detection of these two objects and showcase our adaptive vehicle detection approach.

A variety of features are used extensively in vehicle detection to extract visual features of vehicles, including Haar-like features and HOG features. However, all these methods are affected by the change in environmental conditions to some extent. Even though HOG features are extracted to minimize the lighting and luminance variance by including a normalization stage, the visual features of the vehicle could vary significantly by the change of ambient light from one extreme to the other, i.e., from day to night. This is because the vehicle itself is not a static object with regard to its appearance in different lighting conditions. In other words, since the object’s appearance may change under different environmental conditions, the best approach is to update the detection method based on the ambient light. This is on top of the fact that detecting any object in the dark could be more challenging.

Mostly, the vehicle detection is categorized into two different conditions of light and dark, and different datasets are provided in the literature for these two cases. We consider the third situation of low light where the visual appearance of the object changes extensively, but the vehicle shape features could be considered as an identifying feature and hence could be considered in the classification stage [3]. We name these three different conditions day, dusk and dark. We use different methods to detect the vehicles during these three scenarios. While detection during the day and dusk only differ in their trained model, the detection algorithm for the dark scenario is totally different and results in a completely different hardware accelerator.

We deploy a hardware accelerator for pedestrian detection in parallel with a hardware accelerator for vehicle detection. The vehicle detection hardware accelerator is dynamically updated in the system as the environment lighting changes. At the same time, we maintain uninterrupted pedestrian detection during the system update. The update process is achieved through partial reconfiguration (PR) of the FPGA fabric on Zynq UltraScale+ MPSoC.

PR is an advanced feature of FPGAs, which allows time multiplexing the hardware resources. PR results in having both the flexibility of software implementation and the performance of hardware implementation simultaneously. There are several potential advantages in PR, including but not limited to increasing the effective logic density, saving cost and power by reducing the FPGA for a target functionality, and providing the feasibility of adaptive and more secure systems.

**Figure 1:**
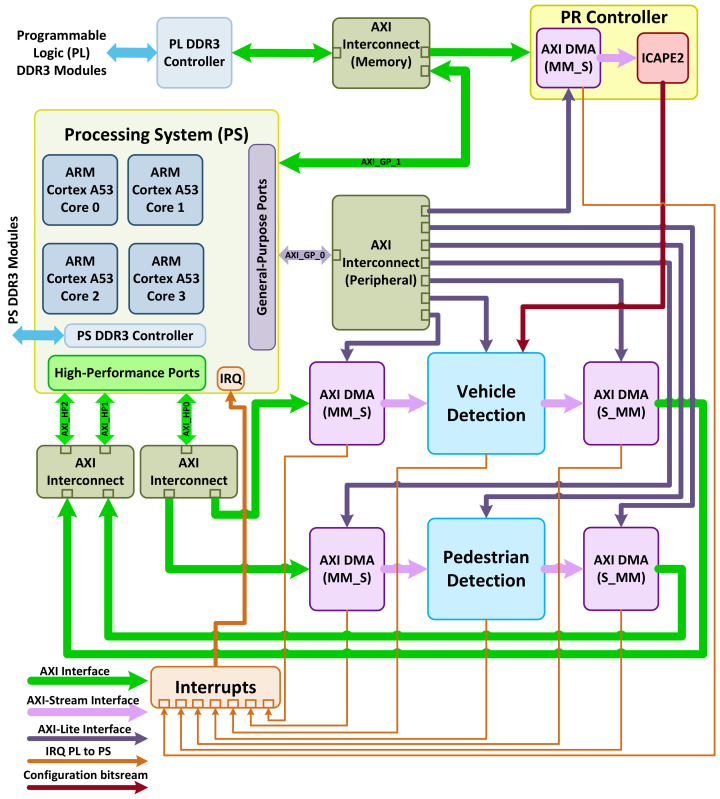
Block diagram of implemented adaptive system.

Figure 1 shows a block diagram of our adaptive system implemented on Zynq UltraScale+ MPSoC. The interface between different parts of the PL and the interface between the PL and PS are maintained through the advanced extensible interface (AXI). The system consists of the pedestrian, vehicle detection hardware accelerators, PR controller on the FPGA fabric and four ARM Cortex A53 processors on the PS side. Both Pl and PS have access to their dedicated DDR3 modules. One processor reads the images from the DDR3 connected to the PS. The image information is then transmitted through high-performance ports on the PS to the PL. These data are received by both the pedestrian and vehicle hardware accelerators through their dedicated direct memory access (DMA) interconnect. Two other processors capture the results of vehicle and pedestrian detection through two separate high-performance AXI interconnects. The other processor is assigned to initiating and monitoring the partial reconfiguration process when the request is generated and triggered by the change in the environment’s lighting condition. While a signal from a light sensor should trigger this process, we emulate this signal for the simplicity of our design.

## 4. Pedestrian and Vehicle Detection

Pedestrian and vehicle detection are the vital tasks in driver assistance systems (DASs) and autonomous driving systems (ADSs). Pedestrian detection is considered one of the most challenging tasks due to the variation in human sizes, poses and their appearance. Reliable detection of pedestrians and humans is also important in several other domains such as video surveillance and robotics.

Vehicle detection is the main and primary task in an ADS, which has been a topic of interest over the last decade. Several research works have studied the detection of cars either based on its appearance, its motion or a combination of both [15]. While motion-based methods look at the sequence of frames and employ both detection and tracking algorithms [16,17], appearance-based detection mainly relies on the pixel information of one image frame. In general, these methods extract vehicle appearance features and compare them with a pre-trained model through the classification stage. Various visual features of vehicles are used for this purpose, including the overall shape of car, edges, corners, underneath shadow of the vehicle, headlight or taillight position and their color. However, all of these features are somehow affected by the environmental condition, which makes the challenge of accurate detection even more complicated.

The DL approaches in object detection achieve more accurate results at the cost of more computation [26]. Although, in some scenarios, the traditional ML approaches might achieve lower detection accuracy than DL-based techniques, these methods are considered good candidates in real-time applications with constrained computation power. The combination of the HOG feature extractor and linear SVM has shown its competency in human and vehicle detection. We base our pedestrian detection and our vehicle detection during the day and dusk on this method. We use DBN-based taillight detection for vehicle detection during the dark and in very low lighting conditions.

### 4.1. HOG+SVM Algorithm

**Figure 2:**
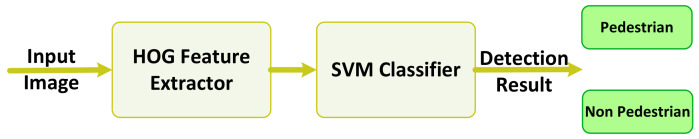
Block diagram of detection based on the HOG feature extractor and SVM classifier.

The principle of object detection in the traditional approach consists of two different stages of feature extraction and object classification. In the first stage, specific features of an image are extracted. In the next stage, the classifier decides whether the object belongs to the particular class based on the calculated features in the initial stage. Figure 2 shows a block diagram of object detection using the HOG feature extractor and SVM classifier. In this method, the HOG features of the input image are first calculated and then passed to the SVM classifier. The classifier compares the HOG features of input with its pre-trained model data, which represents the human model in this case. The result of the classifier defines if the image belongs to the pedestrian class or not.

**Figure 3:**
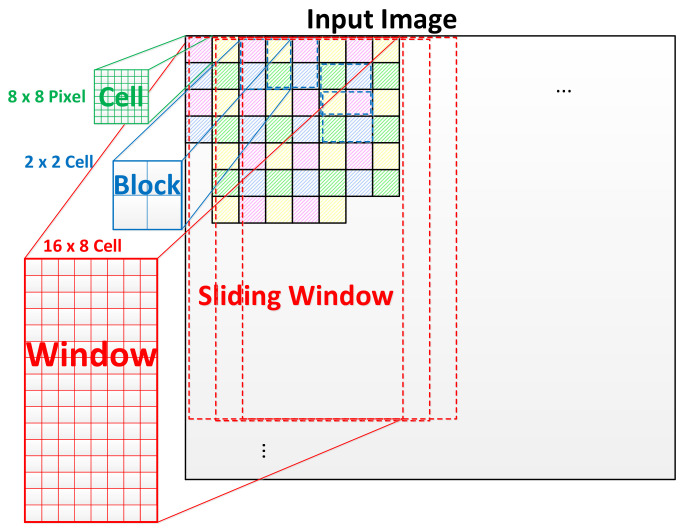
Cell, block and sliding window in detection algorithm based on HOG and SVM. Detection window of 64 × 128 pixels, equivalent to 8 × 16 cells is used for pedestrian detection [2].

Calculating HOG features incorporates dividing the input image into small parts called cells, normally 8 × 8 pixels, as shown in Figure 3. Then, the gradients in both *x* and *y* directions are calculated for each pixel within the cell. Simple [−1,0,1] and ${\left[-1,0,1\right]}^{T}$ gradient filters are applied to the pixel value f(x,y) in order to obtain both $f_{x}\left(x,y\right)$ and $f_{y}\left(x,y\right)$, which are defined as

(1) $$f_{x}\left(x,y\right)=f\left(x+1,y\right)-f\left(x-1,y\right)$$

(2) $$f_{y}\left(x,y\right)=f\left(x,y+1\right)-f\left(x,y-1\right)$$

The gradient magnitude m(x,y) and gradient direction θ(x,y) are then computed as

(3) $$m\left(x,y\right)=\sqrt{f_{x}^{2}\left(x,y\right)+f_{y}^{2}\left(x,y\right)}$$

(4) $$\theta \left(x,y\right)=\arctan\frac{f_{y}\left(x,y\right)}{f_{x}\left(x,y\right)}$$

The gradient histograms are then generated for each cell within the image. The interval of [0,π) is evenly divided into nine bins. This value chosen as the number of orientations is offered by Dalal and Triggs [8] to result in better human detection. The association of a bin with each pixel is based on the value of θ(x,y) and is weighted by the value of m(x,y). The two nearest bins to the value of θ(x,y) are updated to avoid aliasing effect.

The normalization process is the final step in HOG feature extraction. The blocks are the overlapping number of adjacent cells and usually consists of four cells, i.e., a set of 2 × 2 neighboring cells [8]. Feature vectors of the four cells within a block are accumulated to generate a normalization factor. This factor depends on the normalization scheme adopted for this step. We use the *L1-sqrt* normalization scheme, where ϵ is a small constant and *L1-sqrt* is defined as

(5) $$\mathrm{L1-sqrt}:\;\;\sqrt{\frac{\nu }{\left(∥\nu {∥}_{1}+\epsilon \right)}}$$

With ν as the unnormalized feature vector, ${∥\nu ∥}_{k}$, known as the *k-norm* of the vector, is defined as:

(6) $${∥\nu ∥}_{k}={\left(\sum_{i=1}^{n}{\left|x_{i}\right|}^{k}\right)}^{\frac{1}{k}}$$

where $x_{i}$ are the feature vectors, and *n* is the number of feature vectors.

Once the features are extracted and normalized, a window of generated features are passed to the classifier to evaluate the presence of a specific object, i.e., human in the case of pedestrian detection. The concept of overlapping blocks and a sliding detection window is depicted in Figure 3.

The classification stage is based on using an SVM classifier. The linear SVM is a discriminative binary classifier, which is defined by the hyper-plane separating positive and negative regions. Given the training data together with their class labels, the SVM constructs an optimum hyper-plane by its support vectors, which define either a specific set of features belongs to a class of objects or not [9]. In its simplest form for two-dimensional feature space, the SVM generates a line to divide positive and negative samples. SVM classifier looks for the answer of Equation (7) in a way that minimizes *w* so that *E*(*w*), the total hinge loss, is minimized. *w* and *x* are the weight vector and feature vector, respectively, and *n* is the number of elements in the weight vector and feature vector.

(7) $$E\left(w\right)=\frac{\lambda }{2}{∥w∥}^{2}+\frac{1}{n}\sum_{i=1}^{n}max\left\{0,1-y_{i}\langle w,x\rangle \right\}$$

During the detection and at the classification stage, the linear SVM classifier compares the test data with the model data by calculating the dot product of the features vector *x* and the weight vector *w*. The weight vector *w* is calculated and obtained during the training stage. A bias value *b* is also calculated during the training stage and is used in Equation (8) during the classification.

(8) y(x)=w·x+b

The resulting *y*(*x*) defines whether the feature vector belongs to the specific class of objects or not by checking its sign as

$$\left\{ \begin{array}{cc} y(x)>0⟹positive & \left(9\right)\\ y(x)<0⟹negative & \left(10\right) \end{array}\right.$$

The detection accuracy of a classification is defined in Equation (11). The number of correctly detected objects are called true positives shown by *TP*. The number of images that are correctly classified as non-object are called true negatives and is shown by *TN*. *FP* stands for the number of false positives, which are the images incorrectly classified as the object. Similarly, *FN* is the number of false negatives, which are the images that include the objects but are not classified correctly.

(11) $$Accuracy=\frac{TP+TN}{TP+TN+FP+FN}$$

### 4.2. HOG+SVM Models for Pedestrian and Vehicle Detection

**Figure 4:**
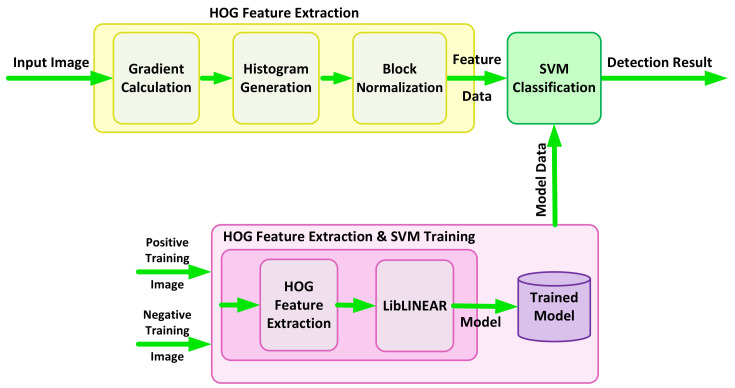
Detection method by HOG+SVM for pedestrian detection, vehicle detection during the day and vehicle detection during the dusk.

Figure 4 shows a block diagram of the training and test procedure. HOG features of an input image are extracted through three main stages of gradient calculation, histogram generation and block normalization, as explained earlier in this section. The features are then classified against a pre-trained model in the SVM classification stage to provide the final results. An SVM model is created during the training phase, where HOG features are extracted for all the training images of a dataset and trained by LibLINEAR [27].

The first step in the implementation of the pedestrian detector is choosing a suitable dataset. We use the INRIA pedestrian dataset [28], which has 2416 pedestrian images, called positive images, and 1218 images where there is no presence of a human in them, known as negative images. To create a richer training dataset, we generate negative images by randomly sampling the negative train images. We generated 12,180 negative train images, which are used in the training process. The test dataset of INRIA consists of 1126 positive images and 453 negative images. With the same random sampling method applied to negative train images, we generate 4530 negative test images to evaluate our classifier performance.

During the day and dusk lighting conditions, where the edge and shape boundaries of vehicles are reliable identifying features, we use the HOG+SVM detection method, as explained in [3]. For detection during the day, a subset of the UPM vehicle dataset [29], which includes a subset of images extracted from the Caltech dataset [30] and the TU Graz-02 dataset [31], is used. The vehicle images in this dataset are aligned with the conditions that we have defined for vehicle detection during the day. In moderate lighting conditions—considered dusk conditions—the SVM classifier is trained using the images in the SYSU dataset [17], which satisfies our definition of dusk scenario. Even though the dataset is aimed at night vehicles, since the images are taken from near cars and in the urban area with a reasonable level of lighting, their visual appearance is quite visible, and we categorize them into the dusk condition. Table 1 summarizes the details of images that are used for train and test our detection models.

Two different models for two different scenarios of day and dusk are generated. The major difference in these two models is seen in the area where taillights are usually positioned within the 64 × 64 window. The day model is created using the day training dataset and is used as input for the classifier during the detection in day lighting conditions. The dusk model trained by the dusk training dataset aims at detecting the vehicles in the dusk scenario.

The trained SVM models were evaluated, and the results show that using the day model for classification in moderate light results in about 20% decrease in detection accuracy. Using the dusk model and the test set of the SYSU dataset [17] resulted in detection accuracy of 82.37%. The achieved accuracy shows a noticeable decrease compared to the day scenario, which could be expected as the detection in medium to low light is more challenging than in bright conditions. Trying to improve this value, we trained the SVM classifier by applying both the day dataset and dusk dataset at the same time. It is noticeable that the trained models in these three cases look very different. The results of evaluation tests show that the accuracy of detection in dusk improves up to the value of 85.34% with the combined model. This could be justified by noting the fact that the visual appearance of vehicles in the dusk scenario is very similar to their appearance in the day with a slight decrease in the sharpness of edges as well as some added features around the taillights.

**Figure 5:**
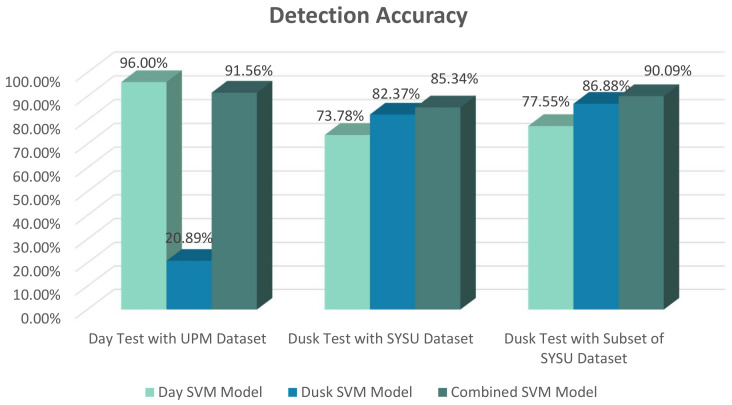
Comparison of accuracy in day and dusk test scenarios with different SVM trained models.

Figure 5 compares the results of different trained models in different test scenarios. It is noticeable that the detection accuracy during the day drops from 96.00% to 20.89% when the dusk model is used instead of the day model. This substantial decrease in accuracy is justified by the fact that the dusk trained model relies mostly on the gradient change around light positions, but the dominant gradient in a vehicle shape in the presence of light happens around the physical boundaries of the car. The detection accuracy during the dusk with the day model eventuates in more tolerable results as it decreases from 82.37% to 73.78%. However, it is still considered as a substantial downfall and is not acceptable. The results confirm the need for two separate SVM models for better accuracy.

### 4.3. Multi-Scale HOG+SVM

We deploy the algorithmic modification to the conventional HOG+SVM multi-scale object detection, which is presented in [2]. In this approach, the normalized HOG features are down-sampled to detect different sizes of the object within an image. By shifting the scale pyramid generation stage to the later stages after the feature extraction, the computational complexity will be reduced significantly. The INRIA person dataset [28] is used to verify the effect of our proposed modification. The original dataset is used to provide an SVM model for pedestrian by training a linear SVM with the extracted HOG features in LibLinear [27]. The model is then used to check the detection accuracy, where a 98.0375% detection rate is obtained over the INRIA test dataset. The original test dataset of INRIA is then up-sampled using the scale value of 1.1 to 2 with the step size of 0.1 to generate a test dataset for humans at various window sizes from 64 × 128 to 128 × 256.

**Figure 6:**
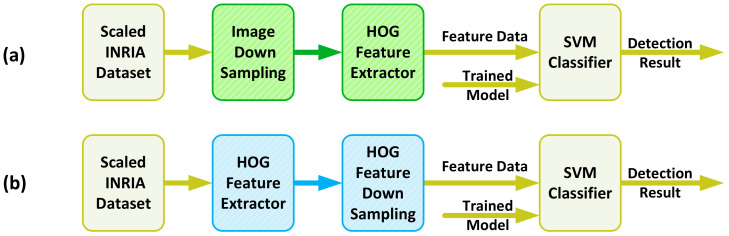
Setup test for two scenarios of (**a**) conventional detection method (**b**) proposed detection method [2].

The verification is accomplished by applying the up-sampled test dataset to two different configurations of the detector, as shown in Figure 6. The first configuration follows the conventional method of HOG+SVM detection, where the image is first resized to the detection window size, and then the features are extracted and fed to the classifier. The second configuration shows our proposed method in which the HOG features are extracted at the first stage, and then the features are resized to match the detection window size equal to the dimension of the trained SVM model.

**Figure 7:**
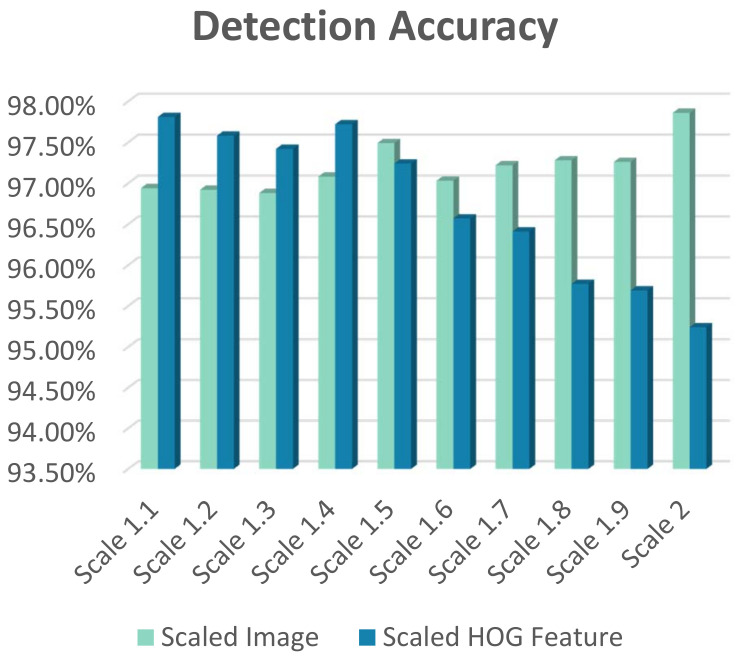
Detection accuracy comparison between the results of the scaled image and scaled HOG features at different scales.

Figure 7 makes the comparison of detection accuracy in the conventional approach and our proposed method in [2] for different scales of image and HOG features. The obtained results for the accuracy shows that at near original scales, up to the scale value of around 1.5, our proposed method outperforms the conventional method, while as the scale value increases from 1.5 to higher values, down-sampled HOG features are not as promising as the resized image.

### 4.4. DBN-Based Vehicle Detection

**Figure 8:**
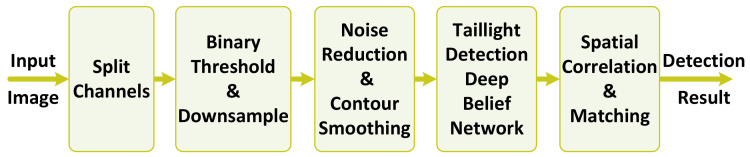
Block diagram of vehicle detection in dark.

The vehicle detection is a more challenging task when the car headlights are the main source of light on the road during the very dark conditions at night. In this scenario, only the objects within a near distance of the light source could be recognized by their shape and visual appearance. In this situation, the vehicle detection mainly relies on the detection of taillights with the expected size, color, and position. We use a two-stage detection method proposed in our other work [3]. During the first detection stage, taillights are detected by a trained DBN. The second stage of detection uses the generated features of the previous stage, which are considered spatially to match the detected taillights and detect the existence of vehicles. Figure 8 shows the block diagram of vehicle detection in the dark. Two preprocessing stages subtract the background and eliminate the noise.

**Figure 9:**
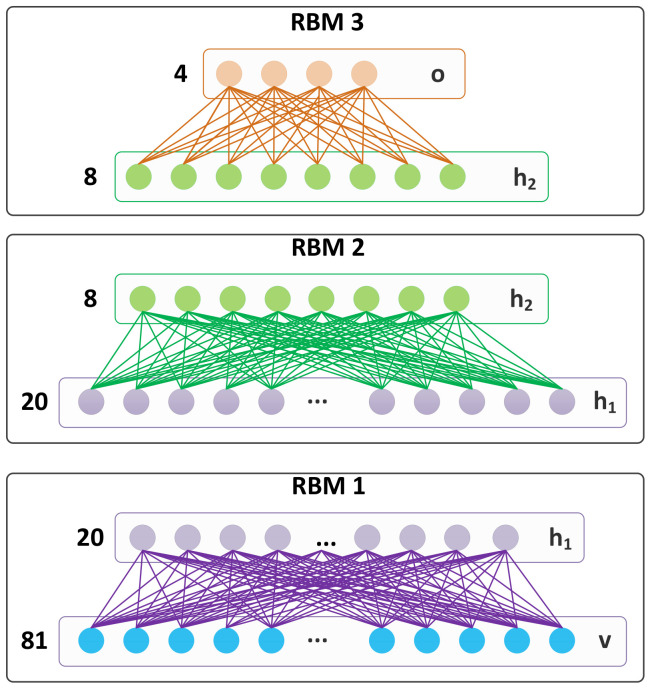
DBN comprised of three separately trained RBMs.

The DBN is trained in Matlab and has 81 visible inputs. These inputs correspond to the binary values of a 9 × 9 window of the image. The architecture of our DBN is shown in Figure 9 shows the architecture of the DBN with two hidden layers with twenty and eight hidden nodes, respectively. The number of hidden layers and their nodes is selected based on the several iterations in training the DBN with different hidden layers and with different node numbers. The current values generate the most promising results with the smallest error rate.

The cropped images of taillights from the training images of the SYSU [17] dataset are used to train the DBN. The final output layer consists of four nodes that determine the presence of taillights with different sizes. Since the distance between the two taillights is expected to be within a specific range, only a particular region around each detected taillight is processed for matching. This will reduce the processing time and increase the reliability of our detection.

## 5. Implementation

**Figure 10:**
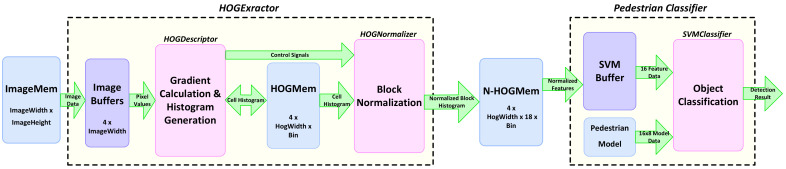
Block diagram of implemented hardware accelerator for pedestrian detection [2].

The hardware accelerator that is used for pedestrian detection and vehicle detection during day and dusk scenarios includes two main stages of HOG extractor and SVM classifier, as shown in Figure 10. The HOG feature extraction consists of a few computation stages. Figure 10 shows two different processing stages within the HOG extraction stage. The gradient calculation and histogram generation steps process the pixels within a cell, while the final step of block normalization modifies the generated histograms within a block. Processing the pixels within the cells has a different data access pattern than the normalization stage. This means some intermediate storage elements are required between these two functional blocks. Moreover, the histograms of cells are updated several times during the processing of different pixels within a cell or its neighbor cells. In our hardware implementation, the gradient calculation and histogram generation are merged into one module named *HOGDescriptor*. Block normalization is kept as a separate module named *HOGNormalizer*. Three different memory structures are considered at the start, middle and end of the implemented pipeline to address different data access requirements of each processing stage. The input image is read from off-chip memory and is passed through a line buffer, *ImageBuffers*, where four rows of the image are stored. This is because three rows of pixels are required for the calculation of $f_{y}\left(x,y\right)$. The other row is updated with new data that are required for the calculations in the next row of pixels.

As discussed above, memory access could impose latency in the processing pipeline, especially in the case of off-chip memories. Furthermore, with the potential resource constraints for on-chip memories and the power/energy overhead, considerations should be taken into account for the wise and efficient use of storage elements. Defining a memory hierarchy that is specifically designed to address the requirements of specific access patterns within an algorithm is beneficial.

The generation of histograms starts with gradient magnitude and direction calculation. The image pixels are read starting from the top left of the image, and gradient values are calculated. The image is scanned through its rows. Since the pixel information from three consecutive rows is required for calculating the gradient, we use four line buffers in the *ImageBuffers* to access and reuse the data needed during gradient calculation. One additional line buffer is considered to be updated while the gradient values in one row are calculated.

The floating point calculations in the *HOGDescriptor* are replaced by fixed-point calculations, which is a common practice in hardware implementations. Moreover, trigonometric functions are implemented through the use of lookup tables (LUTs). $f_{x}\left(x,y\right)$ and $f_{y}\left(x,y\right)$ gradients are calculated in parallel, followed by the calculation of the gradient magnitude and bilinear interpolation within the pipeline. The implemented architecture has an initial delay of around 100 clock cycles and satisfies the timing requirements at the frequency of 300 MHz. However, the calculated gradient values should update the histograms of four adjacent neighbor cells, which requires the possibility of simultaneous access to the memory elements that are storing these updated values. This could potentially put constraints on the throughput if not handled properly within the memory hierarchy.

**Figure 11:**
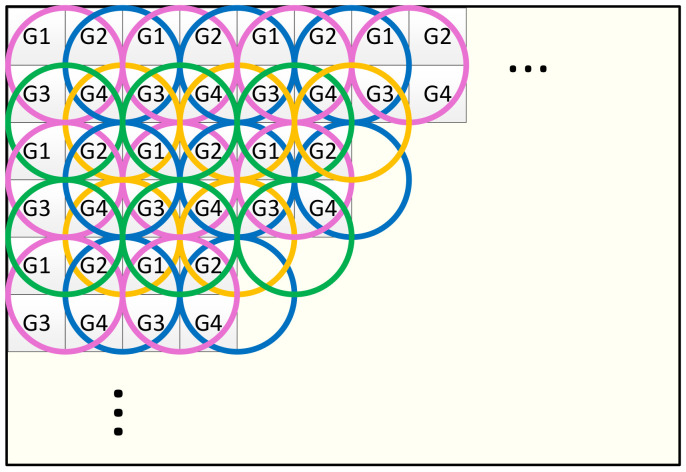
Division of cells into four different groups of G1–G4 results in simultaneous access to all cells contributing to each HOG block [1].

The *HOGMem* is updated by the *HOGDescriptor* and accessed by the *HOGNormalizer*. For an optimal result, memory access requirements at both ends should be taken into account so that the feasibility of a parallel and high throughput processing pipeline is obtained. Figure 11 shows how defining four different groups of cells results in all the blocks containing only one cell from each group. Consequently, assigning separate memory blocks to each group’s cells addresses the requirement of simultaneous access to the cells and that their histograms are updated by calculating one gradient value.

The cell division pattern represented in Figure 11 also guarantees that each block during the normalization stage consists of cells belonging to different memory groups. Consequently, the *HOGNormalizer* has simultaneous access to the data of these memories during the calculation of normalized histograms.

**Figure 12:**
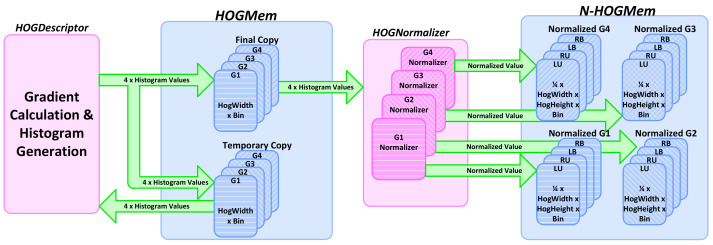
Memory hierarchy of *HOGMem* and *N-HOGMem*. The *HOGMem* consists of two copies updated by *HOGDescriptor*; one of them is accessed to update the gradient values, and the other is used in calculating the normalized version of the histograms. Four instances of memory are considered for each group cell in the *N-HOGMem* and are labeled based on their relative position in the block [1].

We define a similar pattern for the memory storing the results of the *HOGNormalizer*, called *N-HOGMem*. As shown in Figure 11, each cell contributes to four different blocks, taking four different relative positions in four different blocks. As a result, the normalizer generates four different versions of a cell histogram based on the normalization factors calculated for all four blocks that contain a specific cell. In this case, four memory banks are associated with each group from *G1* to *G4* to store four different versions of the cell histogram. Figure 12 shows the structure of *HOGMem* and *N-HOGMem* and their relation with the processing blocks within the *HOGDescriptor*.

*HOGMem* is an intermediate memory storing the un-normalized histograms. The final values of the HOG descriptor are stored in *N-HOGMem*. Consequently, the size of *HOGMem* is maintained as low as possible. The normalization of each row of HOG blocks is completed before the new row of cell histograms are updated completely in *HOGMem*. Consequently, *HOGMem* should only store four rows of cell histograms to minimize the memory resource utilization.

Parallel and pipelined architecture is considered in the implementation of *HOGNormalizer* as well to avoid the addition of any extra delay to the processing pipeline. The calculated normalization factor of a block is used for normalizing all cells within the block at the same time in parallel. This approach results in data reuse and saves both power and time.

As shown in Figure 10, the last stage in pedestrian detection is the classification of HOG features through the linear *SVMclassifier*. The access pattern at this stage differs from the order it is generated at the *HOGNormalizer* side. Therefore, the use of intermediate memory, *N-HOGMem* is inevitable. We show how the choice of compatible pipelined architecture for *SVMclassifier* helps in minimizing the size of *N-HOGMem* memory.

The classifier requires both the normalized feature data from *N-HOGMem* memory and the model data to calculate the dot product of them. The trained pedestrian model is stored in a separate memory and accessed by the *SVMclassifier*, as shown in Figure 10. Each detection window for pedestrian detection is 16x8 blocks, and each block has a 36-element feature vector.

*N-HOGMem* accesses 16 memory banks in parallel to read 16 different HOG features. Even though 16 simultaneous data could be accessed in a cycle, these features do not provide one full column of the detection window. However, in two cycles, one feature for two columns of the window could be obtained from *N-HOGMem*. The SVM classifier obtains the feature vectors of two columns every 72 clock cycles by circling through four different categories of the feature data groups, i.e., *LU*, *RU*, *LB* and *RB*, shown in Figure 12. This is equivalent to accessing the feature vector of one column every 36 clock cycles when the buffers are full.

**Figure 13:**
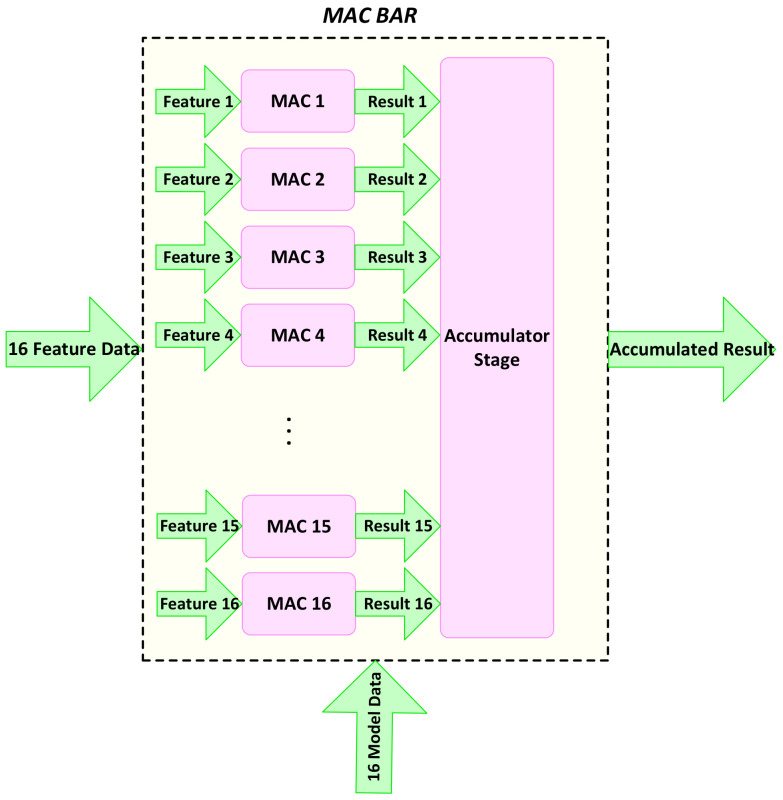
The *MACBAR* parallel architecture consisting of 16 *MAC* units [2].

A parallel architecture matching with memory access is defined for the processing units in the *SVMclassifier*. Balancing the processing speed at both feature extraction and classification helps eliminate extra storage elements. The data features for one column of the window are accessed and fed to the classifier. At the same time, the dot products are calculated by 16 different MAC units that are responsible for the multiplication and accumulation required in the dot product. We name this processing unit *MACBAR*. Figure 13 shows the MACBAR parallel architecture, which consists of 16 MAC units working in parallel, each fed with a model datum and data feature separately.

**Figure 14:**
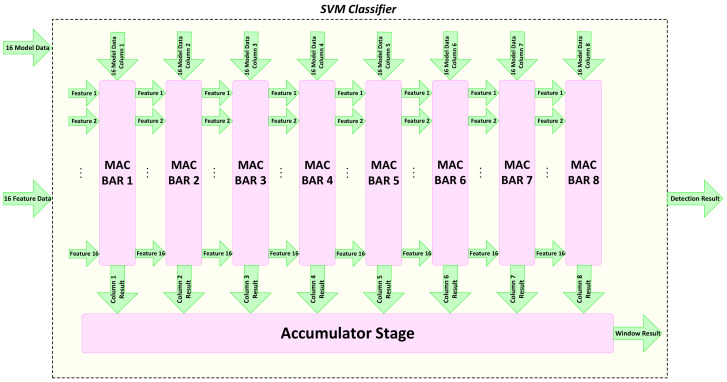
The parallel and pipeline architecture of *SVMclassifier* with 8 parallel *MACBAR* computation unit [2].

Figure 14 shows the implemented architecture of the SVM classifier with eight parallel *MACBAR* computation units. The feature data are fed to the classifier and pipelined through eight stages to calculate eight columns of eight different windows. Consequently, once all eight *MACBAR* units are filled with the data, the classifier calculates the SVM result of a window through 36 cycles. The detection window slides horizontally through the image until it reaches to the end of the row, when a new window starts from the next row. This approach results in a classification speed exceeding the rate of feature extraction. This guarantees that the limited size of *N-HOGMem* considered in the design fully addresses our requirements.

The same hardware architecture is used for the implementation of vehicle detection during day and dusk. The only difference in these implementations is the size of the detection window, which is 64 × 64 for the vehicle detection. The SVM classifier keeps the same hardware architecture and processes two detection windows in 36 clock cycles, which results in double the processing speed compared to the classifier speed in pedestrian detection. However, since the HOG feature extraction stage requires more processing time, this speed-up does not affect the final throughput.

### 5.1. Multi-Scale Detection

Accurate and fast detection of pedestrians is one of the most challenging tasks of an ADS. Humans of various sizes appear on the road at different distances from the car, which results in the detection requirement of considering different sizes. Slight changes in human size are considered within the training stage by feeding variations of positive training samples to the SVM classifier. However, the classifier searches for a specific size of a human within its defined window size. Consequently, the presence of objects with a bigger or smaller size, which do not fit in the detection window, is not achieved through the detection method shown in Figure 2. By using down-sampled images at different scales, the detection of the objects with a bigger size or farther distance to the car will become possible.

The image pyramid is generated by down-sampling the original image by various factors consecutively. The main parts of the detection pipeline, including both HOG feature extraction and SVM classification, is then applied to each of the scaled images separately. The final result is achieved by merging all the detection results and choosing the detection with the highest probability based on the confidence score, as generated by the classifier. The last part is usually handled by the non-maximum suppression (NMS) algorithm [32]. NMS considers the results of all windows that have an overlap of more than a specific value and then choose the window with the highest classification result to represent the final detection.

**Figure 15:**
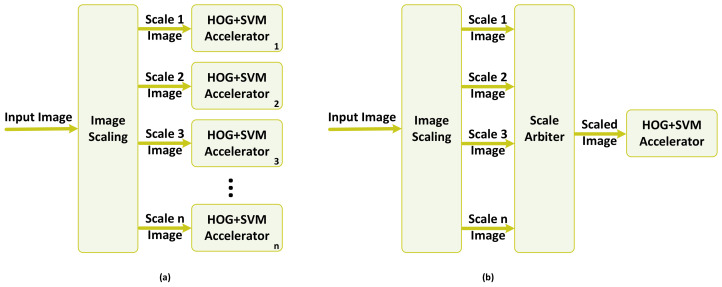
Potential hardware implementations based on the conventional multi-scale detection approach: (**a**) the fully parallel approach; (**b**) the sequential approach.

The real-time requirements of pedestrian detection in safety-critical applications, such as an ADS, require the employment of hardware accelerators within the detection pipeline. In the multi-scale detection scenario, where several scales of the image should be processed to check the presence of an object, the utilization of a hardware accelerator could be conducted in two different ways or a combination of both. Figure 15 shows two different ways of instantiating the hardware accelerator, which processes the HOG feature extraction and SVM classification.

In the first approach, shown in part (a), a functional block of image scaling reads an input image and generates various scales by down-sampling the original image. These scaled images are then processed in parallel by having several instances of the hardware accelerator working in parallel. This approach helps in maintaining the high throughput and real-time performance of the detection at the cost of higher resource utilization and, consequently, higher power/energy consumption.

The second approach, as depicted in part (b) of Figure 15, maintains the resource utilization as low as possible. The task of processing scaled images is handled by the same hardware accelerator, which is in charge of processing the original image. The *ScaleArbiter* functional block circulates through different images in the image pyramid and employs the same hardware accelerator by time multiplexing the access of different scales of the image to it. This approach benefits from low resource utilization; however, it lacks the capability of providing real-time performance and throughput as the processing time is increased by the factor of *n*.

A combination of these two approaches could be used based on the requirements and constraints of the detection system. However, neither of them provides a good solution for sophisticated real-time applications, where both resource and time constraints are critically crucial. We base our hardware implementation on the algorithmic modification discussed in Section 4.3.

**Figure 16:**
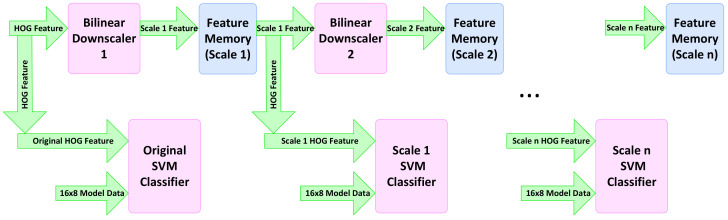
Multi-scale classification using a series of pipelined down-scaling modules [2].

Figure 16 shows the implemented pipeline for the multi-scale classification by down-scaling the HOG features. We employ the memory architecture explained in the previous section and keep the same hardware for the calculation of HOG features to avoid any bottleneck in memory access. The interface between the HOG descriptor and SVM classifiers is achieved through a few instances of feature memories for different scales. These memories have the same architecture as *NHOGMem*, as explained in Section 4.2. These memories provide the data for both the bilinear down-scaler of the next scale as well as the current-scale SVM classifier.

### 5.2. DBN-Based

**Figure 17:**
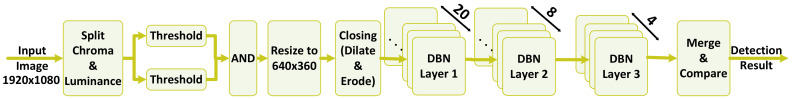
Pipeline of implemented vehicle detection in dark [3].

The implemented pipeline for vehicle detction in dark is shown in Figure 17. Parallel and pipelined architecture is employed within each stage of the processing to maximize the throughput and eliminate extra resource utilization. Vivado HLS high-level synthesis tools are used with the HLS OpenCV library for the hardware implementation of the dark algorithm.

The results of Vivado HLS synthesis for vehicle detection in the dark show that the latency of 2,122,251 cycles with the initiation interval of one cycle is achieved at the frequency of 300 MHz. Consequently, an HDTV frame of 1920 × 1080 is processed within less than 8 ms.

## 6. Evaluation and Comparisons

Dedicated hardware accelerators that are discussed in Section 5 are integrated together to develop an adaptive system that is capable of reconfiguring itself dynamically during the transitions between different lightning conditions. The partial reconfiguration (PR) controller explained in our other work [3] is deployed on Zynq UltraScale+ MPSoC to provide adaptability to the system with minimum reconfiguration time overhead. Zynq UltraScale+ MPSoC includes four ARM Cortex A53 cores. With our hardware-software co-design approach, one of these cores is responsible for initiating the dynamic reconfiguration when the trigger signal is received. Our PR controller has the throughput of 390 MB/s [3] and utilizes negligible hardware resources on FPGA.

The combination of parallel and pipelined architecture with a specific memory pattern definition results in achieving higher throughput for HOG feature extraction and SVM classification in our pedestrian and hardware detection accelerators. Our HOG+SVM accelerators working at 300 MHz on Zynq UltraScale+ MPSoC are capable of processing an HDTV frame in less than 9 ms. This translates to the processing speed of 110 fps.

Table 2 presents the resource utilization of our implemented hardware accelerators. Detailed resource utilizations for three hardware accelerators are provided in the table, along with the final resource usage of the implemented adaptive system. It is evident that by taking a dynamic reconfiguration approach, the resource utilization for three different versions of vehicle detection algorithms is limited to the required resources for one implementation only. Moreover, the PR controller utilizes less than 1% of the available hardware resources on Zynq UltraScale+ MPSoC.

For the purpose of evaluation, we use the performance event counters available on ARM Cortex A53 performance monitor unit (PMU) to measure the number of cycles required for pedestrian detection, multi-scale pedestrian detection and vehicle detection in a 1920 × 1080 image. We use the OpenCV open source library in our C/C++ code and run the program in Linux. The required time for processing one HDTV frame is measured by ARM Streamline. It takes around 1.8 sec for one frame to be processed by the HOG extractor and SVM classifier.

**Figure 18:**
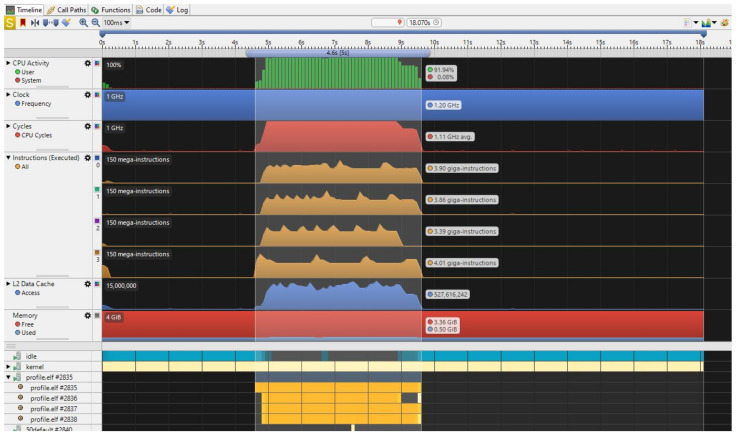
The profiling result for multi-scale pedestrian detection with a scale factor of 1.05 on an HDTV frame running on Zynq UltraScale+ ARM CortexA53 quad-core.

For the evaluation of speeding up the multi-scale detection, the C/C++ implementation is run on the ARM processor available on Zynq UltraScale+ MPSoC with different values of scale ranging from 1.05 to 1.125. The results of profiling using the cycle counter from ARM PMU is shown in Figure 18. The results show that with the scale value of 1.05, it takes 5.0 s for an HDTV frame to be processed. The processing time decreases to around 2.4 s as the scale value increases to 1.125.

**Figure 19:**
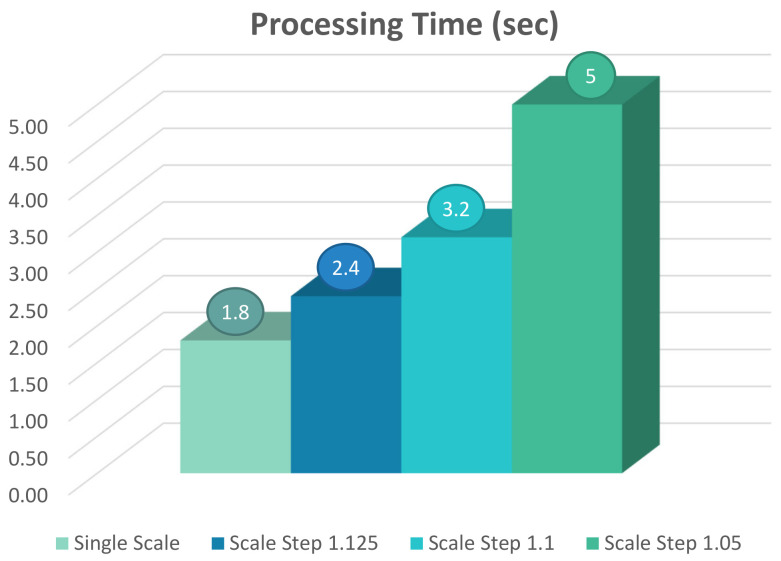
Comparison of ARM processing time for multi-scale detection of pedestrian in an HDTV frame with different scale steps.

The chart in Figure 19 shows how increasing the number of down-sampled images in the detection affects the processing time of a frame. While single-scale detection takes only 1.8 s to finish its task on the ARM processor, having multi-scale detection with the scale step of 1.05 increases the processing time to 5.0 s while utilizing all the four available cores. This value is about three times higher than the time required by single-scale detection, which utilizes only one core.

## 7. Conclusions

This work presents an approach to improving the performance of embedded systems in charge of data processing and decision making in an ADS. This goal is addressed by introducing practical approaches to the development of hardware accelerators for computationally intensive object detection tasks. A special memory hierarchy tailored to the requirements of the algorithm at different processing stages is used in the development of the HOG+SVM detection pipeline. Hardware implementation of the vehicle detection in the dark is achieved by use of Vivado HLS tools. These implemented hardware accelerators are then used as part of the final adaptive ADS through the hardware/software co-design. Implemented work on the Zynq UltraScale+ MPSoC platform provides high-performance and real-time detection. Our work shows that the adaptability of the system is achieved through minimal time and resource overhead.

While our implemented system is mostly based on conventional ML algorithms, the results show a significant improvement in the throughput between the hardware implementation and the pure software implementation running on the ARM processors of the Zynq UltraScale+ MPSoC. The detection accuracy of the algorithms deployed in this work is reasonable but lower than the accuracy obtained in many newly developed deep learning object algorithms. At the same time, with considerably lower resource utilization, this presented work can achieve a high throughput of 110 fps. Moreover, adaptive detection by means of dynamic and partial reconfiguration is applicable to any traditional or deep learning ML algorithms.

## Figures and Tables

**Table 1:** Details of training and test datasets. Test images of INRIA dataset [28], UPM dataset [29] and SYSU dataset [17] are used for test of pedestrian, day and dusk condition respectively.

SVM Model		Training Dataset			Test Dataset		
Name	Size	Image Size	Positives	Negatives	Image Size	Positives	Negatives
**Pedestrian**	7 × 15 × 36	96 × 160	2416	12,180	70 × 134	1126	4530
**Day**	7 × 7 × 36	64 × 64	800	975	64 × 64	200	25
**Dusk**	7 × 7 × 36	64 × 64	450	1345	64 × 64	1063	752

**Table 2:** Resource utilization of hardware accelerators and final adaptive design on Zynq UltraScale+ MPSoC.

Utilization	LUT	FF	BRAM	DSP48
**Available**	274,080	548,160	912	2520
**Adaptive ADS**	46%	28%	32%	21%
**Pedestrian Detection**	16%	9%	13%	1%
**Vehicle Detection-Day/Dusk**	15%	9%	11%	1%
**Vehicle Detection-Dark**	27%	18%	14%	20%
**PR Controller**	0.75%	0.55%	0.27%	0%

## Data Availability

Not applicable.

## References

[1] Hemmati M., Biglari-Abhari M., Berber S., Niar S. HOG Feature Extractor Hardware Accelerator for Real-Time Pedestrian Detection. Proceedings of the 2014 17th Euromicro Conference on Digital System Design.

[2] Hemmati M., Biglari-Abhari M., Niar S., Berber S. Real-Time Multi-Scale Pedestrian Detection for Driver Assistance Systems. Proceedings of the 54th Annual Design Automation Conference 2017 DAC’17.

[3] Hemmati M., Biglari-Abhari M., Niar S. Adaptive Vehicle Detection for Real-time Autonomous Driving System. Proceedings of the 2019 Design, Automation Test in Europe Conference Exhibition (DATE).

[4] Chen A.T.Y., Biglari-Abhari M., Wang K.I.K. (2020). Fusing Appearance and Spatio-Temporal Models for Person Re-Identification and Tracking. J. Imaging.

[5] Viola P., Jones M. Rapid object detection using a boosted cascade of simple features. Proceedings of the 2001 IEEE Computer Society Conference on Computer Vision and Pattern Recognition, CVPR 2001.

[6] Freund Y., Schapire R.E. (1995). A Decision-theoretic Generalization of On-line Learning and an Application to Boosting. Proceedings of the Second European Conference on Computational Learning Theory, EuroCOLT ’95.

[7] Viola P., Jones M.J., Snow D. Detecting pedestrians using patterns of motion and appearance. Proceedings of the Ninth IEEE International Conference on Computer Vision.

[8] Dalal N., Triggs B. Histograms of oriented gradients for human detection. Proceedings of the 2005 IEEE Computer Society Conference on Computer Vision and Pattern Recognition (CVPR’05).

[9] Vapnik V., Golowich S.E., Smola A. (1996). Support Vector Method for Function Approximation, Regression Estimation, and Signal Processing. Advances in Neural Information Processing Systems 9.

[10] Hsu S., Wang Y., Huang C. Human Object Identification for Human-Robot Interaction by Using Fast R-CNN. Proceedings of the 2018 Second IEEE International Conference on Robotic Computing (IRC).

[11] Nikouei S.Y., Chen Y., Song S., Xu R., Choi B., Faughnan T.R. Real-Time Human Detection as an Edge Service Enabled by a Lightweight CNN. Proceedings of the 2018 IEEE International Conference on Edge Computing (EDGE).

[12] Zhu H., Qi Y., Shi H., Li N., Zhou H. Human Detection Under UAV: An Improved Faster R-CNN Approach. Proceedings of the 2018 5th International Conference on Systems and Informatics (ICSAI).

[13] Oren M., Papageorgiou C., Sinha P., Osuna E., Poggio T. Pedestrian detection using wavelet templates. Proceedings of the IEEE Computer Society Conference on Computer Vision and Pattern Recognition.

[14] Brunetti A., Buongiorno D., Trotta G.F., Bevilacqua V. (2018). Computer vision and deep learning techniques for pedestrian detection and tracking: A survey. Neurocomputing.

[15] Sivaraman S., Trivedi M.M. (2013). Looking at Vehicles on the Road: A Survey of Vision-Based Vehicle Detection, Tracking, and Behavior Analysis. IEEE Trans. Intell. Transp. Syst..

[16] O’Malley R., Jones E., Glavin M. (2010). Rear-Lamp Vehicle Detection and Tracking in Low-Exposure Color Video for Night Conditions. IEEE Trans. Intell. Transp. Syst..

[17] Chen L., Hu X., Xu T., Kuang H., Li Q. (2017). Turn Signal Detection During Nighttime by CNN Detector and Perceptual Hashing Tracking. IEEE Trans. Intell. Transp. Syst..

[18] Lecun Y., Bottou L., Bengio Y., Haffner P. (1998). Gradient-based learning applied to document recognition. Proc. IEEE.

[19] Girshick R., Donahue J., Darrell T., Malik J. (2014). Rich Feature Hierarchies for Accurate Object Detection and Semantic Segmentation. Proceedings of the 2014 IEEE Conference on Computer Vision and Pattern Recognition, CVPR’14.

[20] Girshick R. (2015). Fast R-CNN. Proceedings of the 2015 IEEE International Conference on Computer Vision (ICCV), ICCV ’15.

[21] Ren S., He K., Girshick R., Sun J., Cortes C., Lawrence N.D., Lee D.D., Sugiyama M., Garnett R. (2015). Faster R-CNN: Towards Real-Time Object Detection with Region Proposal Networks. Advances in Neural Information Processing Systems 28.

[22] Redmon J., Divvala S.K., Girshick R.B., Farhadi A. (2015). You Only Look Once: Unified, Real-Time Object Detection. arXiv.

[23] Smolensky P. (1986). Information processing in dynamical systems: Foundations of harmony theory. Parallel Distrib. Process.

[24] Hinton G.E., Osindero S., Teh Y. (2006). A Fast Learning Algorithm for Deep Belief Nets. Neural Comput..

[25] Sabour S., Frosst N., Hinton G.E. Dynamic Routing Between Capsules. Proceedings of the 31st International Conference on Neural Information Processing Systems.

[26] Ortiz Castelló V., Salvador Igual I., del Tejo Catalá O., Perez-Cortes J.C. (2020). High-Profile VRU Detection on Resource-Constrained Hardware Using YOLOv3/v4 on BDD100K. J. Imaging.

[27] Fan R., Chang K., Hsieh C., Wang X., Lin C. (2008). LIBLINEAR: A Library for Large Linear Classification. J. Mach. Learn. Res..

[28] INRIA Person Dataset. http://pascal.inrialpes.fr/data/human/.

[29] Arróspide J., Salgado L., Nieto M. (2012). Video analysis-based vehicle detection and tracking using an MCMC sampling framework. EURASIP J. Adv. Signal Process..

[30] The Caltech Database. http://www.vision.caltech.edu/html-files/archive.html.

[31] Opelt A., Pinz A., Fussenegger M., Auer P. (2006). Generic object recognition with boosting. IEEE Trans. Pattern Anal. Mach. Intell..

[32] Rothe R., Guillaumin M., Gool L.V. (2014). Non-maximum Suppression for Object Detection by Passing Messages Between Windows. Asian Conference on Computer Vision ACCV 2014.

